# Preliminary results of a network meta-analysis on the efficacy of long-acting injectable antipsychotics in schizophrenia

**DOI:** 10.1192/j.eurpsy.2021.433

**Published:** 2021-08-13

**Authors:** R. Medrano, F. Carranza, E. Saucedo, A. Guerrero

**Affiliations:** Psychiatry, Centro de Neurociencias Avanzadas UANL, Monterrey, Mexico

**Keywords:** Depot Antipsychotics, schizophrénia, EFFICACY, network meta-analysis

## Abstract

**Introduction:**

Long-acting injectable antipsychotics (LAIs) are currently the most effective alternative for patients with schizophrenia who exhibit poor adherence. Although a recent meta-analysis reported similar efficacy between first and second-generation LAIs, these results were only based on 3 studies due to the limited number of head-to-head comparisons.

**Objectives:**

Present the preliminary results of a network meta-analysis on the comparative efficacy of LAIs in schizophrenia.

**Methods:**

Studies were obtained from a previous study, where we carried out a systematic search from until May 2019 in various databases. Included trials of adults with schizophrenia compared the efficacy of LAI vs LAI or placebo through the Positive and Negative Syndrome Scale (PANSS). Efficacy was evaluated through the mean differences (MD) from baseline to endpoint in the PANSS total scores. Network meta-analysis was performed in MetaInsight through direct and indirect comparisons using a Bayesian approach.

**Results:**

from 12 studies are presented in Figures 1 and 2. All LAIs except zuclopenthixol were more effective than placebo. There were no significant differences between LAIs except for aripiprazole and risperidone, which were more efficacious than zuclopenthixol. The largest change occurred with aripiprazole LAI, but was not significantly higher than haloperidol.

**Figure:**
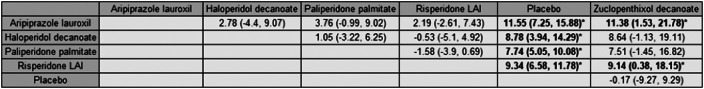

Figure 1. Comparison of treatment pairs. Effect sizes are presented as MD and 95% confidence intervals (*p<0.05).

**Figure:**
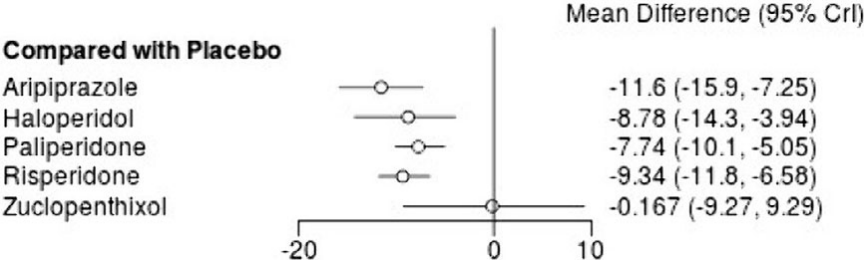

Figure 1. Overall change in symptoms

**Conclusions:**

Preliminary results from a network meta-analysis also suggest that in the long-term haloperidol decanoate is equally effective in overall symptom changes compared to other LAIs. Further analyses are needed to obtain a better perspective on these drugs.

**Disclosure:**

No significant relationships.

